# Molecular Mechanisms Against Successful Weight Loss and Promising Treatment Options in Obesity

**DOI:** 10.3390/biomedicines13081989

**Published:** 2025-08-15

**Authors:** Zsolt Szekeres, Eszter Szabados, Anita Pálfi

**Affiliations:** 1 Department of Laboratory Medicine, Medical School, University of Pecs, H-7624 Pecs, Hungary; szekeres.zsolt@pte.hu; 2 Division of Preventive Cardiology and Rehabilitation, 1st Department of Medicine, Medical School, University of Pecs, H-7623 Pecs, Hungary

**Keywords:** obesity, weight loss, metabolic adaptation, metabolic memory, GLP-1 agonists

## Abstract

**Objectives**: Obesity has become a major health issue, with multifactorial etiologies involving lifestyle, genetic, and neuroendocrine mechanisms. Despite public health campaigns and lifestyle interventions, long-term weight loss is often difficult to achieve or sustain. This literature review aims to summarize current knowledge on the main molecular mechanisms that hinder weight loss and to summarize the newest therapeutic strategies targeting obesity. **Methods**: The literature review was conducted using PubMed, Scopus, and Web of Science databases, with a preference for peer-reviewed original articles, systematic reviews, and meta-analyses. Eligible studies were required to be published in the English language and within the last ten years (2015–2025), with the exception of historically significant publications. A total of 112 articles were included in our review. **Results**: Obesity is a complex, chronic, recurrent metabolic condition that requires personalized, multidisciplinary treatment approaches. In this review, we summarize the major molecular mechanisms underlying weight gain and weight maintenance in obesity. In this literature review, we address the metabolic memory and epigenetics that act through DNA and histone modifications and micro interfering RNAs, resulting in an energy imbalance that can be passed on to further generations. The dysfunction of adipose tissue contributes to chronic low-grade inflammation and insulin resistance, leading to more severe obesity. The ratio of white, beige, and brown adipocytes also plays an important role in regulating energy balance. Novel medical interventions offer promising results in attenuating these mechanisms against successful weight loss. **Conclusions**: Current interventions, including calorie restriction, physical activity, and pharmacological treatment together, may show great promise in combating obesity, but long-term efficacy and safety remain to be established.

## 1. Introduction

Obesity became one of the most pressing public health challenges of the 21st century, as it has reached epidemic proportions worldwide. According to the latest data by the World Obesity Atlas 2025, the prevalence of adult obesity is projected to double between 2010 and 2030, from 524 million to over 1.13 billion [1]. This number is expected to reach almost 2 billion by 2035 if the current trends continue [2]. This surge is not limited to high-income countries, as the middle-income countries are experiencing the most rapid increase. These changes are driven by dietary shifts, sedentary lifestyles, and urbanization. Obesity is the leading risk factor for noncommunicable diseases (NCDs). The World Health Organization (WHO) estimated that obesity led to 1.6 million premature deaths directly in 2021 through cardiovascular diseases (CVDs), diabetes, and cancer [3]. The Global Burden of Disease Study reported a significantly higher number using a comparative risk assessment model: approximately 3.71 million deaths were attributable to high body mass index in the same year, through indirect contributions to mortality [4].

The development of obesity can be related to the imbalance between energy intake and expenditure, as it is a key element in maintaining normal body weight. The composition and quantity of food consumed play a significant role in the development of obesity. On the other hand, energy expenditure, adequate physical activity, the thermic effect of food, and body composition, especially the proportion of skeletal muscle, also largely determine body weight [5]. If the balance between energy intake and expenditure is permanently disrupted, overweight and then obesity develop.

Despite widespread public health campaigns and weight loss strategies, including calorie-restricting diets and physical activity programs, successful weight reduction in obesity remains hard to achieve and even harder to sustain. Evidence shows that even after a successful weight loss, a significant proportion of the weight is regained within a few years [6]. Moreover, the same caloric deficit can lead to vastly different weight loss outcomes across individuals, thus highlighting the complex biological, genetic, and environmental factors that regulate body weight [7].

Evidence has shifted our understanding of obesity from a purely behavioral issue to a more complex, multifactorial disease. Several molecular mechanisms are known that actively work against weight loss and promote weight regain. These mechanisms include genetic causes, neuroendocrine regulations, the amount and the composition of the adipose tissue, and, newly, the metabolic memory of visceral adipose tissue, which is based on epigenetic effects. Epigenetic regulation is a set of environmental influences (e.g., food, exercise, drugs, toxins, psychological influences) that do not change the nucleotide sequence of DNA, but cause changes that affect gene function (e.g., DNA methylation, histone acetylation and methylation, telomerase activity, microRNA changes). Epigenetic changes induce the expression of genes associated with metabolic diseases and inflammation [8].

Recently, some pharmacological treatments have been introduced for the treatment of obesity that have proven effective in achieving weight loss. However, it remains questionable how long an ideal body weight can be maintained after treatment. The aim of this literature review was to explore the latest scientific evidence on the genetic, metabolic, and neuroendocrine barriers to successful weight loss. In addition, this review seeks to provide a structured overview of current lifestyle-based and medical interventions.

To properly and effectively manage this multifactorial disease, we need an adequate, multifactorial approach. In our review, we aim to provide a comprehensive, up-to-date overview regarding obesity management and possible future research directions.

## 2. Materials and Methods

This literature review was conducted using multiple scientific databases, including PubMed (Available online: https://pubmed.ncbi.nlm.nih.gov/, accessed on 9 July 2025), Scopus Available online: https://www.scopus.com/pages/home, accessed on 9 July 2025, and Web of Science (Available online: https://www.webofscience.com/wos/woscc/basic-search, accessed on 9 July 2025), with a preference for peer-reviewed original research articles, systematic reviews and meta-analyses. Eligible studies were required to be published in the English language and within the last ten years (2015–2025), to ensure the inclusion of up-to-date and relevant evidence, with the exception of historically significant publications. The following keywords and phrases were used in various combinations: “weight loss”, “weight regain”, “thrifty gene”, “set point theory”, “metabolic adaptation”, “epigenetics and obesity”, “adipose tissue dysfunction”, “energy expenditure”, “obesity pharmacotherapy”, and “GLP-1 receptor agonist”. All relevant studies meeting the inclusion criteria were considered. Exclusion criteria included conference abstracts, editorials, commentaries, and non-peer-reviewed materials. The identified evidence was organized thematically and, where applicable, chronologically, to reflect the progression of scientific knowledge and the evolution of interventions. A total of 112 articles were included in our review.

## 3. Discussion

### 3.1. Mechanisms of Energy Expenditure

50–65% of total energy expenditure is provided by the basal metabolic rate (BMR), which is the energy required to maintain biological processes during complete rest in a controlled climate environment for at least 10–12 h after the last meal; that is, the minimum amount of energy required to maintain life processes [9]. TEF (thermic effect of food) refers to the energy required to break down and absorb food, which provides 5–15% of energy expenditure. This energy is required for the digestion, absorption, processing, and storage of food [10]. TEF can increase basal metabolic rate when consumed in large portions rather than small, frequent meals, when carbohydrates and protein are consumed rather than fats, and when a low-fat, plant-based diet is followed [11]. The remaining energy expenditure comes from the energy released during any physical activity (TEA, thermic effect of activity), which can vary greatly depending on the activity of the individual. TEA refers to skeletal muscle activity associated with maintaining body position and posture, as well as skeletal muscle activity during physical exercise such as walking, running, swimming, and any other physical activity during the day, like climbing stairs, vacuuming, etc. [9]. Thus, TEA is a highly variable component. Average physical activity accounts for 20–40% of total energy expenditure, but this depends greatly on the individual’s physical activity [12].

The energy consumption of skeletal muscle and adipose tissue is different. The difference between the energy expenditure of skeletal muscle and adipose tissue is 13 kcal/kg/day and 4.5 kcal/kg/day for adipose tissue, respectively [13]. Skeletal muscle has a threefold greater energy expenditure than adipose tissue, so BMR is higher in individuals with high muscle mass, even if their weight is the same as that of someone with low muscle mass. During aging, fat increases at the expense of fat-free mass (FFM); furthermore, loss of lean body mass (LBM) due to sarcopenia (loss of skeletal muscle) and an increase in adipose tissue result in a lower BMR [14].

**Figure 1:**
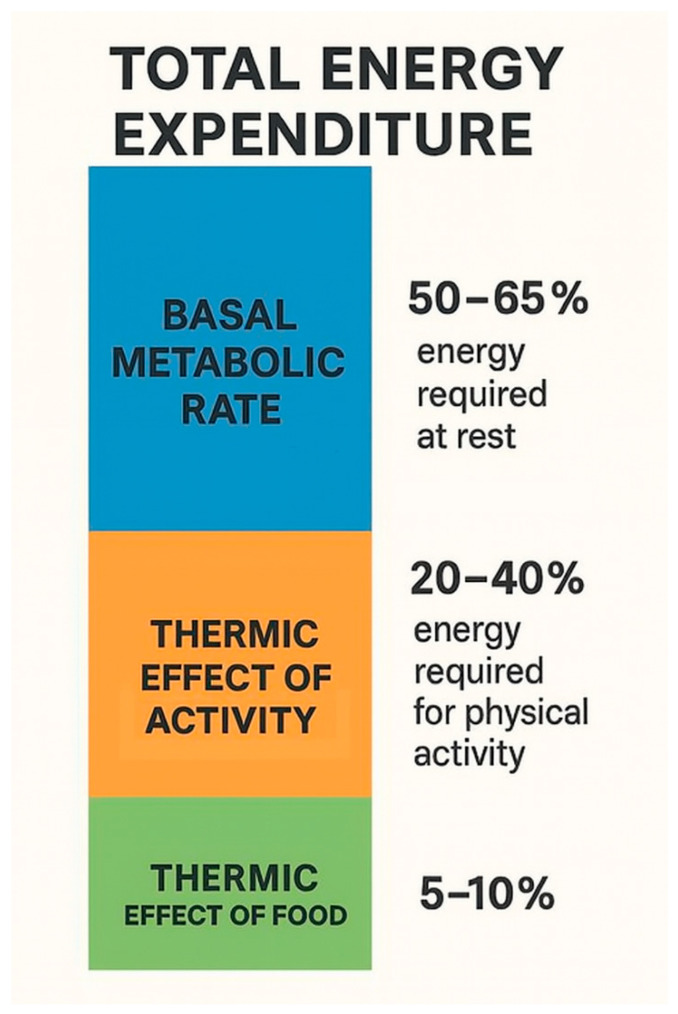
The components of total energy expenditure.

Meal timing can also affect the BMR and energy expenditure. Late eating can lead to a decline in total energy expenditure and core body temperature, which indicates an overall reduced metabolic rate. Late eating also alters gene expression, as it downregulates lipolysis-related genes (PLD6, DECR1, ASAH1, ABHD5), while it upregulates adipogenesis-related genes (GPAM, ACLY, AACS, CERK). Changes can be observed regarding p38, Mitogen-Activated Protein Kinase (MAPK), Transforming Growth Factor Beta (TGF-β), autophagy, and tyrosine kinase signaling, which all favor increased fat storage. These data underline the importance of aligning food intake with personal circadian rhythm, as it can play a significant role in metabolic regulation and obesity risk (Figure 1) [15].

### 3.2. Thrifty Gene Hypothesis

The thrifty gene hypothesis was first proposed by James V. Neel in 1962 [16]. According to this hypothesis, human evolution favored genotypes capable of storing energy efficiently during periods of caloric abundance to enhance survival during times of famine. These “thrifty genes” promote fat accumulation and reduce energy expenditure, providing a selective advantage in prehistoric environments, where periods of food scarcity were more common. In contrast, modern life is characterized by caloric excess and reduced physical activity. These traits thereby predispose individuals to obesity, type 2 diabetes mellitus, and other metabolic abnormalities. Neel originally formed this hypothesis to explain the high prevalence of type 2 diabetes mellitus (T2DM) and obesity in certain populations, particularly among indigenous groups undergoing rapid lifestyle transitions. Although the hypothesis has been influential in shaping early obesity research, it has been widely criticized for its oversimplification of the complex biological mechanisms behind energy homeostasis and for the lack of direct genetic evidence supporting the existence of these specific genes [17]. It has also been challenged on the grounds that famines may not have been universal or prolonged enough to drive such a strong evolutionary selection for energy-conserving genotypes [18]. Despite its limitations, the thrifty gene hypothesis remains a crucial concept, as it introduced a foundation for the research on understanding obesity, while also inspiring alternative theories and more complex models on energy metabolism and weight regulation [19]. Furthermore, the thrifty gene hypothesis may provide a theoretical basis for interpreting metabolic adaptation observed during weight loss.

### 3.3. Set Point Theory

The set point theory was first discussed in the 1970s [20], although its roots trace back earlier to research on the hypothalamic control of body weight and energy homeostasis in animals during the 1950s [21]. The term “set point” refers to a target value maintained by a regulatory system. The theory proposes that each individual has a biologically predetermined weight range that the body aims to sustain through homeostatic mechanisms. When weight deviates from this set range, either through dieting or overfeeding, the body adjusts appetite, metabolism, and energy expenditure to return to this set point via the hypothalamus, leptin, and other pathways. This regulation may explain why weight loss is often met with increased hunger, reduced resting energy expenditure, and the hormonal changes that promote weight regain [22]. This set point is thought to be encoded in the hypothalamus, particularly within the arcuate nucleus, where anorexigenic (e.g., pro-opiomelanocortin (POMC)/ cocaine- and amphetamine- regulated transcript (CART)) and orexigenic (e.g., Agouti-related peptide (AgRP)/Neuropeptide Y (NPY)) neurons respond to leptin and other adiposity signals [23].

Even though the set point theory has provided valuable arguments for understanding energy balance, its concept of a “fixed” set point has been challenged. Evidence suggests that this set point may be dynamic and can shift upward in response to chronic overnutrition, leading to a “settling point” or “dual-intervention point” model. This model argues that the body operates within a flexible biological range rather than around a strict set value [24]. Furthermore, it is known that leptin resistance and hypothalamic inflammation both impair the physiological satiety signaling, thus leading to a pathologically elevated set point in obesity [24].

### 3.4. Melanocortin System and Hypothalamic Regulation

The melanocortin system plays a pivotal role in the regulation of appetite, body weight, and energy balance through complex pathways in the hypothalamus. Its physiological importance has been identified as early as the 1980s [25]. The melanocortin system is inside the arcuate nucleus of the hypothalamus, where orexigenic AgRP/NPY neurons and anorexigenic POMC/CART neurons are located. The arcuate nucleus is a uniquely positioned part of the hypothalamus, located outside the blood-brain barrier, in a region that allows hormonal communication between the blood flow and the brain. These neurons receive and integrate the peripheral hormonal signals such as adipokines, insulin, and ghrelin. The POMC neurons are located more laterally and anteriorly, while the NPY neurons can be found more medially and posteriorly. Despite their proximity, these neurons are functionally antagonistic, as they are interconnected via local inhibitory synapses: the NPY neurons inhibit POMC neurons via a GABAergic pathway. Inside the arcuate nucleus, POMC is cleaved to produce alpha-melanocyte-stimulating hormone (α-MSH), which is an agonist of melanocortin-4 receptor (MC4R), a G protein-coupled receptor, which is crucial for the energy-balance regulation. These receptors can be found deeper in the hypothalamus, for example, in the paraventricular nucleus. Activating this receptor via α-MSH promotes satiety and increases energy expenditure. Contrary, AgRP is an inverse agonist of MC4R, which increases appetite and reduces energy expenditure. During energy deficiency (e.g., caloric restriction), the AgRP/NPY neuron activity is upregulated, while POMC activity is suppressed, resulting in increased hunger and metabolic efficiency [23]. This mechanism is a homeostatic defense of body weight, which is closely related to the set point theory.

### 3.5. Metabolic Memory

The concept of metabolic memory, or obesogenic memory, was first described among type 2 diabetes patients in 2005, based on a follow-up observation of an original cohort in the Diabetes Control and Complications Trial (DCCT) [26]. This publication first described the persistent metabolic effects long after the glycemic state improvements in type 1 diabetes patients. The term has since been extended to obesity and weight loss, as research has demonstrated that metabolic memory–including obesogenic epigenetic changes–can also affect the adipose tissue. This memory may alter energy expenditure, insulin sensitivity, and appetite regulation, even after a significant weight loss. There is an increasing amount of evidence suggesting that epigenetic mechanisms may play a pivotal role in this memory. The nomenclature refers to inheritable, reversible modifications on gene expression that do not result in DNA sequence alterations but include chromatin structure changes as a direct consequence. These changes include DNA methylation, histone modifications, and the regulation of non-coding RNAs [26].

One of the most extensively studied modifications is DNA methylation, which consists of adding a methyl group to the fifth carbon atom of cytosine, thus resulting in 5-methylcytosine. Normally, methylation in humans is normally restricted to cytosine linked by a phosphate to guanine (CpG site); methylation may also occur in non-CpG sites in lower quantities. The human genomes present approximately 28 million CpG sites, and around 60–80% of these are generally methylated. The regions of the genome with high CpG quantities are also called CpG islands (CGIs). Most of these sites are in gene promoter regions, but they can also occur in gene bodies, offering alternative promoter sites. Contrary to CpG sites, the vast majority of CGIs in the promoter regions are generally unmethylated, so that they can maintain the transcription of the active genes. However, there are locations in the genome where multiple CpGs show different methylation status between phenotypes. These are defined as differentially methylated regions (DMRs). The DMRs are more interpretable and statistically important than individually measured CpGs. This means that DMR is a better biomarker in understanding physiological and pathophysiological processes [27]. DNA methylation regulates gene expression through various mechanisms. It may inhibit transcription factor (TFs) binding to promoters, or on the other hand, TFs may recognize methylated sections, and they might affect chromatin structure, thus increasing transcription. Generally, the hypermethylation of the promoter regions results in gene downregulation, while hypomethylation causes upregulation. Severe hypomethylation may even compromise chromatin structure, leading to diseases [28]. If the delicate equilibrium of DNA methylation is interrupted, it can lead to obesity, type 2 diabetes mellitus, cancer, or various other negative consequences.

The alteration of certain obesity-promoting genes (e.g., FTO and peroxisome proliferator-activated receptor gamma gene (PPARγ)) and anti-obesity genes (e.g., LEP, GLP-1R, and POMC) may be associated with obesity. Studies have shown that the global DNA methylation patterns of the adipose tissue and blood of obese patients showed significant hypomethylation. There are more than 700 obesity-related genes that have been identified, and the expression of some of these genes is regulated through differentially methylated CpG sites [29]. According to Zhao et al., every 0.1% increase in DNA methylation in the SLC6A4 gene promoter region is associated with an increased BMI (0.3 kg/m^2^), body weight (0.16 kg), and waist circumference (0.78 cm) [30].

The methylation can be a consequence of environmental or lifestyle factors and may even be passed on to the next generation. This process is called transgenerational epigenetic inheritance. The underlying cause may be the incomplete erasure of these epigenetic changes during gametogenesis and early embryogenesis [31]. Si et al. Conducted a study on 903 mother and child pairs in an epigenome-wide study from the Boston Birth Cohort, a predominantly low-income minority birth cohort. According to their results, maternal pre-pregnancy overweight or obesity significantly altered DNA methylation in newborn cord blood, suggesting an epigenetic factor in the intergenerational risk of obesity [32]. Furthermore, data show that both maternal obesity and underweight may affect the neonatal epigenome during pregnancy, through an intrauterine mechanism. It is also important to note that weight gain during pregnancy showed a lesser importance [33]. However, physiological changes may also alter methylation: according to Jung and Pfeifer, the DNA goes through a progressive global and gene-specific hypomethylation as individuals age. This leads to both genomic instability and to the silencing of specific genes as well [34]. It is also important to highlight that during cancer, the DNA is globally unmethylated, except for certain tumour suppressor genes, which are hypermethylated, compared to other tissues [35]. The important association between obesity and cancer has been shown in numerous studies; however, the underlying mechanisms are still not fully understood. These epigenetic changes may be either the cause or an outcome of metabolic imbalances.

Histone modifications also play a crucial role in epigenetics. The acetylation of histones is generally associated with gene activation. During this process, histone acetyltransferases add acetyl groups to histones, loosening chromatin structure and thus enabling transcription. Another modification is the histone deacetylation via histone deacetylases, which remove acetyl groups and suppress gene expression. These enzymes are upregulated in obesity at specific gene sites, thus contributing to inflammatory gene expression and insulin resistance [36]. The inhibition of this process in animal models led to improvements in insulin sensitivity and energy expenditure. Histones are also subject to methylation that can lead to both the activation and suppression of genes, depending on the specific site and on the number of methyl groups added [37]. These changes have been identified in the adipose tissue of obese individuals. Data show that during obesity, the promoter region of insulin-sensitive genes has reduced acetylation, which is linked to metabolic dysfunction [38].

Non-coding RNAs, especially microRNAs (miRNAs), play a pivotal role in the post-transcriptional regulation of gene expression. The dysregulation of non-coding RNAs contributes to adipocyte hypertrophy, chronic low-grade inflammation, and impaired lipid metabolism [39]. miRNAs are small RNAs that suppress mRNA translation or even promote degradation. For example, miR-143 is known to promote adipogenesis [40], whereas miR-27a inhibits adipocyte differentiation by downregulating PPARγ [41]. Takanabe et al. have found that high-fat diet-induced obese mice have an increased expression of miR-143 [42], while according to Kim et al., the level of miR-27a in obese mice was down-regulated [41]. These data suggest that lifestyle factors directly affect the levels of certain non-coding RNAs, contributing to metabolic disorders and obesity. These epigenetic changes can affect the genes that are involved in adipogenesis, energy metabolism, inflammation, or even appetite regulation in obesity [43].

**Figure 2:**
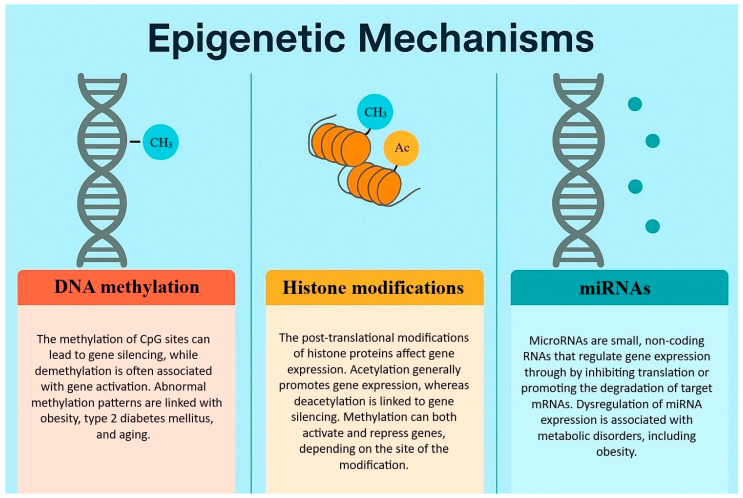
The epigenetic mechanisms that play a crucial role in obesity.

In summary, in our current understanding, it is not clear how epigenetic changes fully affect the metabolic changes observed in obesity. Longitudinal studies and functional characterization studies may help broaden our knowledge regarding these key changes in the future, as they might provide potential targets for future therapies (Figure 2).

### 3.6. Adipose Tissue Dysfunction

Obesity leads to profound structural and functional changes in the adipose tissue. Hyperplasia allows a physiological expansion of the adipose tissue, as it is mediated by progenitor cells. In contrast, adipocyte hypertrophy generally leads to dysfunctional adipocytes that undergo necrosis and contribute to the inflammation of the adipose tissue. The hypertrophy of adipose cells in obesity often exceeds the capacity for adipocyte hyperplasia. This leads to mechanical stress, cellular hypoxia, and recruitment of pro-inflammatory macrophages, resulting in chronic low-grade inflammation and systemic insulin resistance [44]. Fat accumulation in humans can be classified as central obesity, where patients have a high waist-to-hip (WHR) ratio, and peripheral obesity (with a lower WHR). Central obesity poses a severe cardiometabolic risk [45].

Chronic excessive caloric intake leads to adipocyte dysfunction that is associated with quantitative and qualitative changes in the cellular composition of the adipose tissue. One of the most crucial changes in this regard is in the immune response. The chronic low-grade inflammation that is connected to obesity leads to an increase in the total number of immune cells present in the visceral adipose tissue [46]. Hypertrophied fat cells secrete chemokines and undergo necrosis, attracting macrophages into the adipose tissue. Studies show that the number of macrophages in obesity can increase several-fold, producing inflammatory cytokines. In addition to macrophages, an accumulation of pro-inflammatory T-lymphocytes can also be observed. These T-cells can further amplify inflammation by recruiting and activating macrophages [47]. A recent single-cell study of obese human adipose tissue provided a deeper understanding of this inflammatory milieu [48]. It has been shown that weight loss can partially reverse the obesity-related immunological changes, as the adipose macrophage content decreased. On the other hand, the remaining macrophages still expressed a disadvantageous pro-inflammatory gene profile, suggesting an incomplete resolution of the inflammation. This can also mean that the adipose tissue may remain immunologically sensitive in formerly obese individuals, contributing to weight regain or even residual metabolic risk. Also, the local inflammation of adipose tissue does not stay local, as it has systemic effects as well. Visceral fat can deliver inflammatory mediators directly to the liver through portal circulation. As adipose-derived cytokines enter the bloodstream, they may induce a state of chronic, low-grade systemic inflammation [48]. This can be reflected by an elevated level of C-reactive protein and other inflammatory markers in obese patients. Furthermore, it is also known that obesity leads to reduced adipose tissue capillarization, limiting nutrient delivery and contributing to adipocyte dysfunction and insulin resistance [44]. Clinical studies suggest that expending fat may outgrow its own blood supply due to defective angiogenesis, leading to ischemia, hypoxia, necrosis, and inflammation [49]. Also, the inflammation causes fibrosis in the tissue, due to the increase in the synthesis of several extracellular matrix components, especially collagen VI [50], which leads to further impaired metabolic function [51].

The adipose tissue not only stores energy, but it is also recognized as an important regulator of many systemic processes through the secretion of bioactive proteins, referred to as adipokines. The dysfunctional adipocytes produce unbalanced quantities of adipokines, which contribute to the systemic pro-inflammatory state [52]. This inflammatory milieu is characterized by an increased secretion of pro-inflammatory cytokines (such as TNF-alpha, IL-6, and MCP-1), which impair the insulin signaling pathway both locally and systemically. They can also affect cardiovascular risk indirectly by altering the metabolism in the liver, skeletal muscle, and heart. They can promote insulin resistance within the adipose and vascular tissues, contributing to endothelial dysfunction and increased cardiovascular risks. The list of known adipokines is vast; they can be further classified as anti- or pro-inflammatory adipokines. Leptin is one of the most well-known adipokines. Not only does it regulate satiety, but it also suppresses lipogenesis and promotes the breakdown of triglycerides and cholesterol and the oxidation of fatty acids [44]. The dysfunctional adipocytes secrete increased quantities of pro-inflammatory adipokines–such as leptin–and decreased amounts of anti-inflammatory adipokines–such as adiponectin and omentin -, contributing to the escalation of metabolic derangement. These changes are more prominent in the visceral adipose tissue, which explains why visceral adiposity is more significant both in insulin resistance and cardiovascular risk [53]. Furthermore, the elevated levels of leptin are also related to leptin resistance, which is seen both in the central nervous system and in the peripheral tissues. The resistance may result from impaired leptin transport through the blood-brain barrier, chronic inflammation, and disruptions in the leptin receptor signaling pathways. As a consequence, the central satiety-signaling is disrupted, causing hyperphagia and weight gain despite the abundance of leptin [54]. Collectively, these changes all lead to a self-inducing cascade of adiposopathy that poses a significant threat to weight loss.

The attenuation of adipose tissue dysfunction could be a key factor in combating obesity in the future. Thus, future research is warranted for the exact mechanism regarding adiposopathy and how it directly affects the adipokine secretion, potentially providing targets for future therapies.

### 3.7. White, Brown, and Beige Adipose Tissue

Adipose tissue can be found in different subtypes classified as white adipose tissue (WAT), brown adipose tissue (BAT), or beige adipose tissue (BeAT). WAT is primarily responsible for energy storage, but it also plays an important role in the hormone system. These adipocytes contain a single large lipid droplet and only a limited number of mitochondria [55]. In contrast, BAT is a thermogenic organ that is capable of increasing body temperature. For this reason, they contain greater numbers of mitochondria and several smaller lipid droplets. Exposure to cold stimulates BAT and activates thermogenesis through sympathetic pathways [56]. The pivotal molecule in this reaction is the unique thermogenic protein uncoupling protein 1 (UCP1) that can be found in the inner mitochondrial membrane [57]. By uncoupling mitochondrial respiration from adenosine triphosphate (ATP) production, mitochondrial membrane potential is transformed into heat. This process requires a high number of substrates, including intracellular triglycerides and circulating free fatty acids, and glucose [58]. Under the given circumstances, white adipocytes may transform into beige adipocytes. These cells, unlike WAT, contain multiple lipid droplets and UCP1-positive mitochondria to an extent [59]. Also, the UCP1 of beige adipocytes is only induced after specific stimulation; without it, the expression levels are as low as in the white adipocytes [60].

BAT is located in the interscapular, supraclavicular, axillary, and peri-adrenal region in infants. Smaller sites have also been located behind the sternum and along the spine in newborns. BAT is also present in adults, primarily in the cervical, supraclavicular, paravertebral, periaortic, and perirenal sites. The adult human BAT is heterogeneous, as it contains both multilocular and unilocular adipocytes as well. Cold exposure activates these cells, which leads to increased energy expenditure, insulin sensitivity, and lipid clearance [61]. The presence of BAT is negatively associated with cardiovascular risk in obese subjects; however, the exact mechanism remains unknown [62]. Similarly, patients with central obesity have decreased UCP1 expression, suggesting an association between BAT activity and WAT distribution. The proposed mechanisms through which BAT decreases cardiovascular risk include the secretion of BAT-derived adipokines, also called batokines, and the removal of circulating glucose and lipids to improve metabolic health [63].

The exact similarities and differences between brown and beige fat cells are not yet known. Classical brown adipocytes have a higher basal UCP1 expression and elevated uncoupled respiration. Beige cells, however, upon activation, elevate their UCP1 levels to a comparable level. This means that these adipocytes are more likely to be bifunctional, capable of energy storage and heat production as well [60]. It is also known that the loss of brown fat increases the quantities of beige fat in a compensatory way, restoring both body temperature and resistance to diet-induced obesity [64]. However, it is known that the beige adipocytes are located in subcutaneous adipose tissue, and their activity is associated with improved glucose metabolism, enhanced insulin sensitivity, and reduced adiposity. Given this characteristic, these adipocytes are currently considered as a promising target for the treatment of obesity and other metabolic disorders [65]. The regulation of these tissues is not yet fully understood; however, it is already seen that it affects metabolic pathways that are important in obesity as well [63].

Thus, the composition of adipose tissue may directly affect the metabolic state. Even though it is still unclear to what degree this imbalance affects energy expenditure, further studies may indicate a novel therapeutic strategy for obesity in affecting adipocyte function.

### 3.8. Microbiome

The human gut contains trillions of microorganisms that can act collectively as an independent organ. They can affect nutrient absorption, energy balance, inflammation, and even appetite regulation [66,67]. The latest data has confirmed the link between gut dysbiosis and obesity. The alterations in microbiota composition and function have been observed in obese individuals, indicating that these changes can contribute to increased fat storage and difficulties in weight loss [66,68]. For example, Firmicutes bacteria can metabolize polysaccharides into short-chain fatty acids, thus increasing the host’s energy uptake. However, the produced fatty acids, like propionate and butyrate, play a dual role in energy balance, as they can also regulate fat storage and appetite. For example, they may stimulate the release of satiety hormones and enhance fat oxidation. These effects may inhibit adipose fat accumulation and increase energy expenditure. Some Proteobacteria (e.g., Escherichia species pluralis) thrive on high-fat, low-fiber diets and may contribute to inflammation. Similarly, Akkermansia muciniphila also metabolizes fat, which can lead to fat oxidation and satiety. Weight-loss interventions (diets or surgery) can cause a partial shift in microbiome ratio, but some obesity-associated microbial features can persist even after weight reduction [66].

Another key mechanism is metabolic endotoxemia, which means that diets high in fat may increase intestinal permeability, allowing bacterial components like lipopolysaccharides (LPS) to enter the circulation. These derivatives may promote weight gain and insulin resistance. Bacteroidetes are one example of bacteria viewed as a source of LPS, as studies have found elevated levels of LPS in the plasma of obese individuals that correlated with visceral fat mass. The LPS-induced inflammation contributes to insulin resistance, thus creating a cycle of hyperinsulinemia and further increased fat storage [66].

Evidence suggests that after caloric restriction, the post-diet microbiome may remain in an energy-efficient state, predisposing individuals to weight regain when the normal diet resumes [66]. Thus, obesity is characterized by a microbiome that not only reflects the obese state but also actively contributes to its maintenance. For this reason, further research is needed to properly assess the exact link between gut microbiome dysbiosis and metabolic regulation (Table 1).

### 3.9. Lifestyle Interventions

The most common method for weight loss is dietary calorie restriction (CR). It is defined as the reduction of energy intake below ad libitum levels, without leading to malnutrition. It can be expressed as the percentage reduction from baseline intake. It aims to maintain an adequate nutrient intake through the reduction of total caloric intake. Studies have suggested that CR may delay the onset of several age-related diseases (including cardiovascular and neurodegenerative diseases) and may even cause the remission of type 2 diabetes mellitus [69,70]. CR in mice induces beige fat development in the subcutaneous and visceral fat. According to this data, it is possible that CR may lead to the recruitment and activation of BAT [71].

Catechins are natural antioxidants that can be found in green tea. According to a meta-analysis, daily intake of catechin-rich tea in patients increased daily energy expenditure by 5%, as well as fat oxidation [72]. According to Takeshi et al., catechins may even activate BAT and increase energy expenditure [73].

High-fat diet may lead to the hypermethylation of the POMC promoter region, leading to gene suppression, and finally, to increased appetite and suppressed satiety. This methylation can be passed on to the offspring as well [74]. Contrary, certain substances, such as polyphenols (found in fruits and vegetables), catechins (found in tea and coffee), can alter the DNA methylation to a favorable state, thus improving obesity. Similarly, micronutrients, such as B vitamins, folate, and choline, contribute to DNA methylation as methyl donors and co-factors [75]. One of the proposed mechanisms through which nutrients affect DNA methylation is called “one-carbon metabolism”. During this process, one-carbon groups are transferred from donors to proteins or DNA. This involves cyclical chemical reactions, where nutrients such as folate, vitamin B12, and B6, choline, among many others, play a crucial role as co-factors, methyl acceptors, or even donors [76]. The pathway starts with a one-carbon molecule transferred from serine to tetrahydrofolate. After this, a chain reaction follows, catalyzed by enzymes containing B vitamins. The final product is s-adenosylmethionine, a universal methyl donor for methylation processes inside mammalian cells [75]. Dolinov also found that the abnormal hypomethylation of the Avy gene in agouti mice leads to the decreased function of MC4R in the hypothalamus, thus resulting in obesity. If the mice were fed methyl-rich diets (high levels of folic acid and methionine, for example), the offspring’s phenotype showed a beneficial change [77]. CR and weight loss also elevated histone acetylation and improved the expression of glucose transporter 4 in the adipose tissue [78]. This also confirms that epigenetic changes play an important role in obesity, which is affected by dietary factors.

A study has shown that both mouse and human adipocytes retain transcriptional and epigenomic markers of prior obesity post-weight loss, which are associated with faster weight regain upon re-exposure to a high-fat diet [79]. But not only a high-fat diet, but also alcohol intake and smoking can affect DNA methylation. There has been data regarding the association between chronic alcohol consumption and methyl donor nutrient deficiency, which leads to altered one-carbon metabolism. Since alcohol may inhibit methionine synthase activity in the liver, it may lead to a significant decrease in the levels of s-adenosylmethionine [80]. Tobacco smoking also interferes with the one-carbon cycle, as hydrocarbons originating from tobacco smoke are capable of interacting with folic acid and vitamin B12, leading to their biological inactivation [81].

Healthy diets, such as the Mediterranean diet, have been associated with advantageous metabolic changes in obesity. According to novel data, it may affect even the gut microbiome composition as well [82]. Gut microbiota-derived metabolites play a crucial role in metabolic pathways throughout the body. For example, short-chain fatty acids derived from bacterial fermentation of non-digestible dietary fibers may directly affect adipose tissue metabolism. They may modulate lipid buffering capacity, adipokine secretion, insulin sensitivity, and even inflammation [83]. Thus, the restoration of the physiological gut microbiome may attenuate the disadvantageous metabolic alterations seen in obesity.

Physical activity is still one of the most effective non-pharmacological interventions for both the prevention and treatment of obesity. It is a pivotal component of increasing energy expenditure. It has several beneficial effects: it improves cardiovascular health, improves insulin sensitivity and glucose tolerance, increases WAT mitochondrial activity, decreases the levels of circulating lipids, thus reducing the risk of obesity, T2DM, and other metabolic diseases [84]. It is associated with the decrease of the secretion of pro-inflammatory, and with the increase of anti-inflammatory adipokines [85]. Exercise promotes the browning of WAT, enhancing mitochondrial biogenesis and thermogenesis [86]. Moreover, physical activity involves changes in epigenetic modifications. Exercise interventions have been shown to modify obesity-associated DNA methylation patterns, potentially reversing some of the unfavorable epigenetic programming in adipose tissue [87]. These beneficial epigenetic changes are not limited to methylation only, as they affect histone acetylation and miRNA expression as well, which all improve metabolic flexibility and may even counteract the metabolic memory associated with obesity [88].

### 3.10. Pharmacological Interventions

Recent developments in the treatment of obesity have led to several mechanisms through which we are already capable of attenuating obesity. These drugs include β3 adrenergic receptor (β3-AR) agonists, glucagon-like peptide-1 receptor agonists (GLP-1RA), and other agents (such as metformin, tirzepatid, naltrexone, and bupropion).

Several antidiabetic therapies can result in weight loss in varying degrees. Metformin therapy for T2DM is associated with a modest reduction in weight through the modulation of appetite and the AMPK pathway [89]. The sodium-glucose cotransporter 2 inhibitors (such as empagliflozin and dapagliflozin) also induce weight loss through various mechanisms. Clinical data report a 2–4 kg weight loss on average in the first few months of therapy [90]. Furthermore, data suggest that empagliflozin may decrease leptin resistance, thus attenuating adipose tissue dysfunction [91].

It is known that short-term exposure to cold activates brown/beige fat tissue and improves insulin sensitivity [92]. For this reason, cold mimetics like nonspecific sympathomimetic drugs emerged. Since sympathetic receptors are expressed in numerous locations, their clinical applications are limited due to cardiovascular risks. β3-AR is expressed in rodent brown adipocytes, where it plays a role in lipolysis and thermogenesis [93]. Whether the β3-AR is the predominant isoform expressed in human BAT is still unclear; still, promising efforts have been made to develop β3-AR agonists to treat metabolic disorders. Mirabegron is a selective β3-AR agonist, which was approved by the United States Food and Drug Administration (FDA) in 2012 for the treatment of overactive bladder. Several clinical trials have studied it for the treatment of obesity in higher doses. Loh et al. found that a daily intake of 100 mg increased skin temperature and thus energy expenditure in patients without any cardiovascular side effects. However, in higher doses (150 and 200 mg), the blood pressure and heart rate were increased [94]. With the identification of more selective β3-AR agonists, even more precise effects could be achieved in the future.

The GLP-1RAs were originally developed to improve glucose metabolism in patients with T2DM. Since then, additional effects have been observed. Not only do they lower blood sugar levels and improve insulin sensitivity, but they also lead to weight loss, reduce low-grade inflammation, and even have cardioprotective effects. With direct effects both in the hypothalamus and in the gastrointestinal tract, they suppress appetite and delay gastric emptying [95]. It has also been reported that liraglutide, a GLP-1RA, has induced a brown-fat-like phenotype in vivo and in vitro [96]. Furthermore, a recent study conducted by Zhao et al. suggested that liraglutide promoted the remodeling of visceral WAT via regulating miRNAs [97]. A longitudinal study with 25 obese T2DM patients also found that patients treated with GLP-1RAs have elevated energy expenditure rates [98]. These data show that the weight loss observed during GLP-1RA therapy is at least partially due to increased energy consumption. Some studies have reported side effects during GLP-1RA therapy. On one hand, randomized controlled clinical trials and observational studies suggested that there was no significant connection between GLP-1RA therapy and acute pancreatitis [99]. On the other hand, there have been reported cases of acute pancreatitis, and for this reason, the FDA retained a warning for a potential risk [100]. Furthermore, a 2023 meta-analysis found that GLP-1RA treatment was associated with an increase in thyroid cancer [101]. In contrast, a large cohort study reported no significant increase in thyroid cancer risk in patients receiving this therapy. This finding was also reinforced by the American Thyroid Association, highlighting the absence of elevated risk [102]. Clinical guidelines now recommend caution for GLP-1RA treatment in patients with a higher risk of thyroid cancers. GLP-1RA therapy can lead to a 15% weight loss [103]. The GLP-1Ras are available in both once-weekly subcutaneous and once-daily oral or injectable dosage forms.

Another treatment option is the combination of naltrexone and bupropion. Bupropion stimulates hypothalamic POMC neurons, while naltrexone blocks the endogenous opioid-mediated POMC autoinhibition, thus enhancing satiety signaling. This synergism leads to reduced food intake, and it may also modulate the mesolimbic reward pathways. The side effects may include nausea, vomiting, constipation, headache, and dizziness. Available data suggest that this combination could also be used as part of long-term weight loss maintenance [104].

Tirzepatide is a novel, once-weekly injectable dual incretin receptor agonist. It acts on both the GLP-1 and the glucose-dependent insulinotropic polypeptide (GIP) receptors. This leads to enhanced insulin and decreased glucagon secretion, while the gastric emptying and the appetite are both reduced. These effects are significantly more effective than the standalone GLP-1 receptor agonist effect [105]. According to the available research, the therapy results in a 20–22.5% weight reduction in both diabetic and non-diabetic obese patients. Side effects include nausea, vomiting, and diarrhea, especially during dose escalation. Tirzepatide represents a promising pharmacological alternative with a bariatric-level efficacy [106].

The N-methyl-D-aspartate (NMDA) receptor is a glutamate-activated cation channel. The available data suggest that the glutamatergic transmission and the NMDA receptors are important for body weight homeostasis. Antagonizing NMDA receptors in the rodent brainstem leads to a short-term increase in food intake. In contrast, antagonizing NMDA receptors in the hypothalamus has been associated with a reduction in both appetite and body weight. Prolonged systemic administration can induce anorexia. Furthermore, the antagonists also decrease palatable food preferences in rodents [107] and non-human primates [108] and even reduce binge-eating episodes in humans [109]. These contrary effects on feeding can hinder the weight-lowering efficacy of NMDA receptor antagonists. For this reason, there have been efforts to produce dual molecules. For example, the combination of a GLP-1RA and an NMDA antagonist, bound together through a disulfide linker, has led to a molecule that reduced rodent body weight by 23.2% over a 14-day treatment period (Table 2) [110].

## 4. Bariatric Surgery

Bariatric surgery, particularly Roux-en-Y gastric bypass and sleeve gastrectomy, still represents an effective treatment for obesity. Both result in significant weight loss, though, according to novel data, Roux-en-Y gastric bypass shows significantly higher total weight loss and significant advantages regarding dyslipidemia and gastro-esophageal reflux disease [111]. Moreover, bariatric surgery procedures induce significant and long-term alterations in the gut microbiome as well. These include increased microbial diversity, reduced amounts of Firmicutes, and an increase in Proteobacteria and Akkermansia muciniphila, which may be linked to improved metabolic profiles [112].

## 5. Summary and Conclusions

Obesity is a growing global public health crisis, with prevalence expected to rise even more progressively in the coming decades. In recent decades, it has become clear that obesity is not merely a behavioral problem, rather a multifactorial disease that is influenced by genetic, metabolic, neuroendocrine, and epigenetic factors. While imbalances between energy intake and expenditure are crucial to its development, there are also several data showing more complex underlying mechanisms. For this reason, the lifestyle intervention remains inefficient in achieving and sustaining the ideal body weight.

Energy expenditure is mostly determined by basal metabolic rate, physical activity, and the thermic effect of food. However, body composition, for instance, can also influence the energy balance. The thrifty gene hypothesis suggests that the energy-conserving genes once provided survival advantages in times of great famine, which now predispose to obesity and T2DM in the modern environment. The hypothalamic set point theory takes more factors into consideration, as it suggests that the body is genetically programmed to an ideal weight range. If the weight deviates from it, the body counteracts to return to this set point through compensatory mechanisms. It is known that the melanocortin system in the hypothalamus plays a crucial role in maintaining body weight through POMC and AgRP neurons. These neurons affect appetite and energy balance, which is disrupted in obesity. Furthermore, the metabolic memory, which acts primarily through epigenetic mechanisms, uses DNA methylation, histone modifications, and miRNAs to achieve long-lasting effects on gene expression. This explains why previously obese individuals are more prone to regain the lost weight. The adipose tissue dysfunction promotes inflammation, insulin resistance, and hormonal imbalances. Visceral fat is particularly important in the development of metabolic complications. Also, the differences between white, brown, and beige adipocytes explain why they are potential targets for obesity treatment. Although lifestyle interventions remain fundamental in the treatment of obesity, they alone may not be sufficient for a significant proportion of patients. In these cases, pharmacological interventions can complement lifestyle changes that promote weight loss and improve the person’s metabolic status. However, these therapies should still be used with caution and on an individualized basis, as they might possess side effects. Overall, the long-term management of obesity requires a dedicated, personalized, and multidisciplinary approach, the development of which requires further studies.

## Figures and Tables

**Table 1:** Possible mechanisms regulating weight changes.

Main Mechanisms	Pathways	Description
Decreased energy expenditure	Sedentary lifestyle	Reduced thermic effect of activity
	High-calorie diet (processed foods)	Increased calorie intake
	BMR alteration (disadvantageous muscle/fat ratio)	Lower basal metabolic rate
Genetic predisposition	Thrifty gene	Increased fat accumulation and reduced energy expenditure
	Genetic drift	Removal of natural selection allowed more diverse genetic variations
Cerebral set range theory	Hypothalamic pathway	Through neuroendocrine regulation, the body aims to achieve the set weight range
	Appetite regulation	Through endocrine mechanisms, the calorie intake is increased
	Decreased energy expenditure	Lower basal metabolic rate
Neuroendocrine regulation	POMC	When cleaved, α-MSH is produced, which is an agonist of MC4R, thus increasing satiety and energy expenditure
	AgRP	An inverse agonist of MC4R, decreases satiety and energy expenditure
	NPY	Through the inhibition of POMC neurons they decrease satiety and energy expenditure
	MC4R	Activation through agonists shifts the metabolism to anorexigenic state, while inverse agonists cause orexigenic state.
Metabolic memory/epigenetic alterations	DNA methylation alterations	Hypomethylation of obesity-related genes has been found in obese patients
	Histone modifications	Histone deacetylation via histone deacetylases promotes inflammation and attenuate insulin sensitivity
	miRNA regulation	The dysregulation of miRNA function leads to adipocyte hypertrophy and impaired lipid metabolism
Adipose tissue dysfunction	Increased inflammation	Hypertrophied adipocytes promote inflammation, leading to insulin and leptin resistance
	Altered adipokine secretion	Increased levels of pro-inflammatory, decreased levels of anti-inflammatory adipokines
	Disrupted satiety-signaling	Leptin resistance can lead to decreased satiety and increased calorie intake
Composition of adipose tissue	Brown and beige adipocytes	Bifunctional adipocytes capable not only of energy storage, but also heat production
	UCP1	Allows uncoupled respiration for the adipocytes, thus increasing energy expenditure
Microbiome	Increased ratio of Firmicutes bacteria	May increases the host’s energy uptake
	Elevated number of Bacteroidetes	Increased LPS in the circulation, further decreasing insulin sensitivity

**Table 2:** Pharmacological interventions targeting body weight.

Drug Class	Example	Approved for Obesity?	Weight Loss Effect
Biguanides	Metformin	No	Subtle
β3-AR agonists	Mirabegron	No	Subtle
SGLT2 inhibitors	Canagliflozin	No	Moderate
Opioid antagonist/Dopamine reuptake inhibitor	Naltrexone/bupropion	Yes	Moderate
GLP-1RA	Liraglutide	Yes	Substantial
GLP-1/GIP dual agonists	Tirzepatide	Yes	Substantial
NMDA antagonist/GLP-1RA	Not available	Not available	Substantial

## Data Availability

No new data were created or analyzed in this study. Data sharing is not applicable to this article.

## Abbreviations

The following abbreviations are used in this manuscript:

NCDs	Noncommunicable Diseases
WHO	World Health Organization
CVDs	Cardiovascular Diseases
BMR	Basal Metabolic Rate
TEF	Thermic Effect of Food
TEA	Thermic Effect of Activity
FFM	Fat-free Mass
LBM	Lean Body Mass
MAPK	Mitogen-Activated Protein Kinase
TGF-β	Transforming Growth Factor Beta
T2DM	Type 2 Diabetes Mellitus
POMC	Pro-opiomelanocortin
CART	Cocaine- and Amphetamine- Regulated Transcript
AgRP	Agouti-related Peptide
NPY	Neuropeptide Y
α-MSH	α-Melanocyte Stimulating Hormone
MC4R	Melanocortin-4 Receptor
DCCT	Diabetes Control and Complications Trial
CpG	Cytosine linked by a phosphate to guanine
CGIs	CpG Islands
DMRs	Differentially Methylated Regions
TFs	Transcription factors
PPARγ	Peroxisome Proliferator-Activated Receptor Gamma
miRNAs	microRNAs
WHR	Waist-to-Hip
WAT	White Adipose Tissue
BAT	Brown Adipose Tissue
BeAT	Beige Adipose Tissue
UCP1	Uncoupling Protein 1
ATP	Adenosine Triphosphate
CR	Calorie Restriction
β3-AR	β3-Adrenergic Receptor
GLP-1RA	Glucagon-like Peptide 1 Receptor Agonists
FDA	Food and Drug Administration
GIP	Glucose-Dependent Insulinotropic Polypeptide
NMDA	N-methyl-D-aspartate
LPS	Lipopolysaccharide
